# Effects of Early Bedside Cycle Exercise on Gastrointestinal Function in Intensive Care Unit Patients Receiving Mechanical Ventilation

**DOI:** 10.3389/fmed.2022.823067

**Published:** 2022-06-09

**Authors:** Tingting Yu, Fuliang Cai, Rong Jiang

**Affiliations:** ^1^Intensive Care Unit, The First People’s Hospital of Kunshan, Kunshan, China; ^2^Department of Clinical Nursing, School of Nursing, Nanjing Medical University, Nanjing, China

**Keywords:** early bedside cycle exercise, critical illness, gastrointestinal failure, mechanical ventilation, exercise therapy

## Abstract

**Background:**

A prolonged stay in the intensive care unit (ICU) is associated with gastrointestinal failure, which may have a destructive effect on functional status within 1 year after hospital discharge. The aim was to investigate the effects of a daily exercise session, using a bedside cycle ergometer, on gastrointestinal functions, such as diarrhea, gastric retention, and vomiting, in patients with severe pneumonia who received mechanical ventilation (MV).

**Methods:**

The study was a randomized controlled trial, and its setting was the ICU of a tertiary hospital in Eastern China. A total of 102 critically ill patients who received MV were recruited only when their cardiorespiratory function was deemed stable to perform a bedside cycling exercise. Those patients were expected to spend a minimum of 7 days in the ICU. All subjects received respiratory physiotherapy and performed a daily standardized passive or active motion session of their limbs. The patients were randomized into two groups, namely, the treatment group, which were administered passive or active leg exercise intervention for 20 min/day using a bedside ergometer, and the control group, which did not. Gastrointestinal (GI) functions and the nutritional status of both groups were evaluated on the first, fourth, and seventh days of training and at discharge.

**Results:**

During the 7 days of the study, the number of patients with diarrhea in the treatment group was significantly lower than that in the control group. In contrast, there were significantly more patients in the treatment group with increased bowel sounds (*P* < 0.05). However, there was no significant difference in the number of patients with vomiting and gastric retention between these two groups. Moreover, when the patients were discharged from the hospital, the albumin level and lymphocyte count were significantly higher in the treatment group (*P* < 0.05). In addition, the number of invasive ventilation days in the treatment group was less than that in the control group (*P* < 0.05). While the ICU length of stay and the total hospitalization time were not significantly different between the two groups.

**Conclusion:**

Early exercise training in critical ICU survivors who received MV enhanced the recovery of gastrointestinal functions and improved the patient’s nutrition status at hospital discharge.

## Introduction

Critical illness is the reason that patients in intensive care units (ICUs) are often being treated for serious gastrointestinal (GI) dysfunctions or diseases (1). Different GI problems, including vomiting, high gastric residual volumes, diarrhea, bowel dilatation, and absent bowel sounds, may occur in more than half of the ICU patients receiving mechanical ventilation (MV) (2, 3). Furthermore, feeding intolerance caused by delayed gastric emptying occurs in approximately 50% of critically ill patients (4, 5). Several studies have confirmed that GI symptoms frequently occur in ICU patients, with up to 62% of patients exhibiting at least one GI symptom for at least 1 day (2, 6). Intestinal dysfunction is a predominant determining factor that predicts prognosis in critically ill patients, which may lead to impaired outcomes, such as increased length of hospital stay, delayed recovery, increased morbidity or mortality, and could have a negative effect on the patient’s quality of life after hospital discharge (7, 8).

Even so, there are no methods of curing GI problems that have been met with consensus. Because the incidence of GI symptoms seems to be the highest in the first week of ICU hospitalization, it is very important to prevent or attenuate GI symptoms and complications at the beginning of hospitalization in patients who are expected to have prolonged bed rest (9). Standard treatment guidelines of the European Respiratory Society and European Society of Intensive Care Medicine suggest that active and passive physiotherapy for critically ill patients be performed as early as possible (10). Conducting early mobility therapy after ICU admission has been increasingly accepted (11, 12). Early mobilization in the ICU has been shown to improve gut function and reduce delirium duration, MV time, risk of hospital-acquired infections, and length of stay in both the ICU and the hospital (13–17).

Bedside cycle ergometer training has been regarded as a relatively recent treatment that trains patients who have been exposed to prolonged immobilization (18, 19). This exercise training pattern is deemed to have a safe and feasible influence on long-term bed-bound patients with severe chronic obstructive lung disorders and on patients with end-stage renal failure who are treated by hemodialysis (20). However, determining whether using a bedside cycle ergometer during a daily exercise session, as a treatment alternative, plays a vital catalytic role in improving GI functions has not been previously investigated, especially in patient cohorts that require MV. Therefore, the primary aim of this study was to evaluate whether early rehabilitation using a bedside cycle ergometer had any impact on GI failure in patients in the ICU who required MV.

## Materials and Methods

### Study Design and Setting

The study was a randomized controlled trial that was conducted in the ICU of Kunshan Hospital, a tertiary hospital in Eastern China. The experimental procedures were approved by the research ethics committee of First People’s Hospital of Kunshan and Nanjing Medical University. Written informed consent was obtained from all patients or a close relative of sedated patients. Sedated patients signed their informed consents when they regained consciousness. All procedures and protocols conformed to the standards of use of human participants in research as outlined in the Sixth Declaration of Helsinki.

### Patients and Study Design

The study subjects were enrolled from August 2020 to August 2021. All eligible subjects were males and females who were at least 18 years of age. Patient eligibility for inclusion in the study was judged on the second day of admission to the ICU by the attending intensivist, unrelated to the study. Patients were admitted to the Intensive Care and treated by MV for 1–2 days since they were transferred from the emergency department or wards. Patients who were expected to have a prolonged ICU stay of at least 7 days were eligible for inclusion.

The patients were constantly monitored by an ECG monitoring instrument besides their bed every day, and cardiorespiratory stability was determined based on the clinical judgment of the attending intensivist, unrelated to the study. The occurrence of cardiorespiratory instability or other medical conditions impairing the interventions after inclusion led to the exclusion of the patients. The sample size N1 = 40 cases in the treatment group and N2 = 40 cases in the control group were demonstrated by a difference in the biochemical parameter albumin level with a statistical power of 90% and an alpha level of 0.05. Allowing for a 20% loss to follow up or withdrawal rate, at least 48 subjects per group was desired. In this study, 152 patients were included.

Exclusion criteria were as follows: primary gastrointestinal diseases such as gastrointestinal carcinoma, peptic ulcer, and inflammatory bowel disease (IBD); brain failure; malignant tumor; pregnant women; and contraindications to the training of a bedside cycle ergometer. Patients extubated within 48 h after inclusion in the research study, those with unstable hemodynamics or complications during the operation process, including pneumothorax, pulmonary infarction, deep venous thrombosis (DVT), re-intubation, and unsuccessful spontaneous breathing trials (SBT), were also excluded.

### Interventions

In this randomized controlled trial, sealed opaque envelopes in random block sizes were used to allocate patients to either a treatment group or a control group. This randomization procedure was performed by the attending intensivist unrelated to the study. These two groups were treated with routine respiratory physiotherapy and conventional passive or active rehabilitative training, which improved the motor ability of the legs. Conventional physical therapy was performed by staff physical therapists who had at least 2 years of experience in caring for critically ill patients.

**FIGURE 1:**
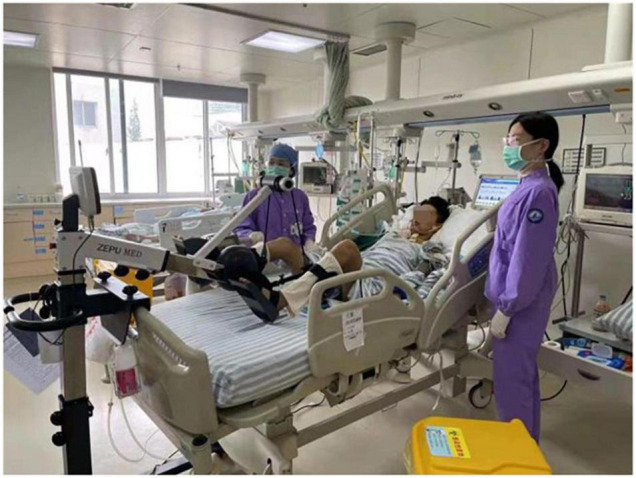
Bedside cycle ergometer (Shandong ZEPU Medical Technology Company, China).

All patients received 20-min physical therapy sessions twice daily. The protocol consisted of passive diagonal movements based on the proprioceptive neuromuscular facilitation stretching technique for the upper and lower extremities and manual bronchial hygiene techniques, such as mechanical vibration sputum expectoration, turnover, and patting back regularly (19, 21). The treatment group, in addition to conventional physical therapy, undertook passive or active cycling exercise training for the lower extremities using a bedside cycle ergometer. The aim of each session was to have the patient cycle for 20 min at an individually adjusted intensity level. The passive or active exercise training of the lower extremities was delivered by a bedside cycle ergometer produced by Shandong ZEPU Medical Technology Company of China (ZEPU-K2000E; Figure 1).

This device was designed to improve resistance based on the patient’s need for an individually adjusted intensity level. During exercise cycling, subjects were placed in the supine position with the headrest elevated approximately 30°–45°. In sedated patients, cycling was performed in a passive manner for 20 consecutive minutes at a fixed pedaling rate of 20 cycles/min once a day in the afternoon prior to daily standardized passive or active physical therapy until extubation (cessation of MV) or day 7 of the protocol. The patients who were able to manage active cycling could switch back and forth between passive and active modes to enjoy uninterrupted exercise. The resistance was zero and the direction was clockwise in passive cycling, while the increasing resistance was between 0 and 20 Nm in active cycling. At every session, training intensity was evaluated and an attempt was made to increase the resistance by one level, as tolerated by the patient. The motor power was 8 watts.

For the cycling exercise, some physiologic parameters were continuously monitored and calculated, such as blood pressure, including systolic and diastolic pressure (SBP and DBP), heart rate (HR), pulse, breathing rate, and percutaneous arterial oxygen saturation (SpO_2_). The experimental procedure was stopped if the parameters of the patients’ physiological response were unstable, such as HR > 100 or < 40 beats per minute, SBP > 180 mmHg, SpO_2_ not more than 90%, or if some other clinical manifestations of cardiorespiratory failure occurred (22, 23).

### Data Collection

Data collection was collected on the day before training, on the third day, and on the seventh day after the patients underwent training. Data collection was repeated on the day before discharge.

The following data were obtained from the patients’ medical records: age, sex, reason for ICU admission, disease severity according to the Acute Physiology and Chronic Health Evaluation (APACHE) II, duration of MV, ICU stay or hospital stay at the time of testing, and GI function abnormalities and nutrition-related indicators. There was no objective and clinically relevant definition of GI dysfunction in critical illness. The Working Group on Abdominal Problems (WGAP) of the European Society of Intensive Care Medicine (ESICM) developed the definition for “GI dysfunction,” which was used to describe the large variety of GI symptoms including diarrhea, vomiting, gastric retention, and increased bowel sounds. The following terminology and definitions used in this study were suggested by the WGAP (6). Vomiting was the occurrence of any visible regurgitation of gastric content irrespective of the amount (24). Diarrhea was defined as having three or more loose or liquid stools per day with a stool weight of more than 200–250 g/day (or greater than 250 ml/day) (25). Gastric residual volume could be considered high if a single volume exceeded 200 ml (26). The normal frequency of bowel sounds may range between 4 and 5 sounds/min, and absent peristalsis was present if no bowel sounds were heard at cautious auscultation, while hyperperistalsis was present if excessive bowel sounds were heard on auscultation (27). Biochemical parameters such as albumin, hemoglobin, and total lymphocyte count (TLC) have long been identified as objective indicators of nutrient malabsorption associated with GI dysfunctions (28–30).

Two attending intensivists blinded to group allocation and intervention provision collected these variables. This was a team of researchers.

### Statistical Analysis

Continuous variables are presented as the mean ± SD or as the median and interquartile range. Two classified variables were presented as absolute and relative frequencies. The Student’s *t*-test was used to compare nutrient parameters between the control group and the intervention group. The Mann–Whitney U test was performed to compare the number of days free from MV and the length of ICU stay and a hospital stay of the patients in the control and treatment groups. The Chi-square test was used to compare the numbers of people suffering from diarrhea, vomiting, and gastric retention. The GI functions of the patients are indicated in numbers and percentages. All statistical calculations were performed using the SSPS software, version 19.0 (IBM Corporation, Armonk, NY, United States). A *P*-value less than 0.05 was considered significant.

## Results

### Patient Flow

**FIGURE 2:**
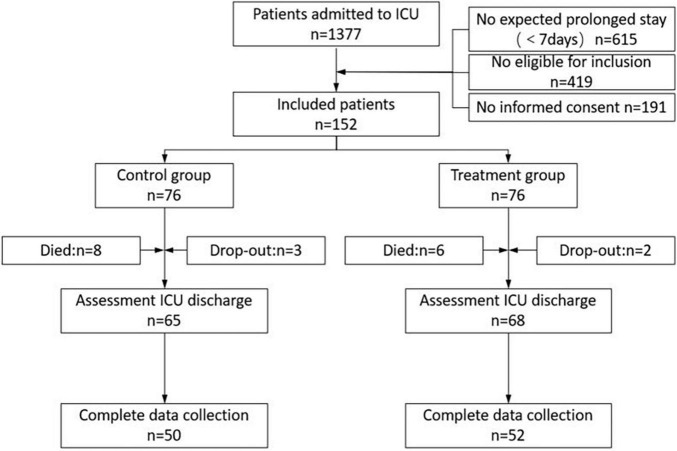
Flow diagram of the patients admitted to this trial.

Figure 2 is a graphical display of the study subjects’ flow. During the enrollment period, 1,377 patients were transferred to the ICU, of whom 615 stayed less than 7 days. A total of 343 patients who were expected to have prolonged ICU hospitalization (7 days) met the entry requirements in the study. Informed consent was unobtainable in 191 patients. A total of 152 consecutive patients were randomly divided into the treatment group (*n* = 76) and the control group (*n* = 76). The mortality rate difference during the ICU stay was not significant in either group (10.5% in the control group vs. 7.9% in the treatment group; *P* = 0.58). In the control group, three study subjects developed cardiorespiratory instability and dropped out, and two subjects in the treatment group dropped out. Incomplete measurements at hospital discharge were present in 15 of the remaining 65 patients in the control group and in 16 of the remaining 68 patients in the treatment group due to an unexpected hospital discharge (for financial reasons). In conclusion, 50 and 52 patients in the control and intervention groups, respectively, were included for analysis.

### Baseline Characteristics and Safety

At baseline, these two groups were similar with respect to demographic, physiological, and ICU diagnoses and treatments (Table 1). There were no significant differences in the baseline data for sex, age, BMI, diseases, APACHE II score, hemoglobin, white blood cells, platelets, and other ventilator treatments.

**TABLE 1:** Baseline characteristics of patients at inclusion in this trial.

	Control group (*n* = 50)	Treatment group (*n* = 52)
Gender, male/female	36/14	38/14
Age, years	69.74 ± 8.13	69.96 ± 8.14
BMI, kg/m^2^	22.97 ± 2.51	23.5 ± 2.78
PaO_2_, mmHg	108.72 ± 29.41	107.02 ± 25.06
PaCO_2_, mmHg	44.62 ± 11.6	42.73 ± 9.86
pH	7.45 ± 0.095	7.44 ± 0.083
Hemoglobin, g/dL	105.81 ± 29.2	110.42 ± 22.31
White blood cells, × 10^9^/L	12.99 ± 5.69	13.31 ± 5.37
Platelet, × 10^9^/L	177.88 ± 60.76	181.92 ± 55.2
APACHE II score on ICU admission	17.84 ± 3.45	16.62 ± 4.1
History of cardiac disease, *n* (%)	7(14%)	11(21.2%)
History of respiratory disease, *n* (%)	13(26%)	11(21.2%)
History of digestive disease, *n* (%)	1(2%)	2(3.8%)
History of hypertension, *n* (%)	22(44%)	29(55.8%)
History of diabetes, *n* (%)	16(32%)	13(25%)
History of cerebral apoplexy, *n* (%)	6(12%)	8(15.4%)

As shown in Table 2, there were also no significant between-group differences in blood gas parameters at inclusion, such as arterial partial pressure of oxygen (PaO_2_), arterial partial pressure of carbon dioxide (PaCO_2_), PH, and oxygenation index (PaO_2_/FiO_2_) (*P* > 0.05). All patients completed the early exercise intervention without any adverse events, as the previous study reported (31).

**TABLE 2:** Blood gas analysis of patients at inclusion.

	Control group	Treatment group		
	(*n* = 50)	(*n* = 52)	|t|	*p*
PaO_2_, mmHg	108.72 ± 29.41	107.02 ± 25.06	0.315	0.753
PaCO_2_, mmHg	44.62 ± 11.6	42.73 ± 9.86	0.887	0.377
pH	7.45 ± 0.095	7.44 ± 0.083	0.516	0.607
PaO_2_/FiO_2_(P/F)	250.72 ± 44.66	243.39 ± 43.23	0.831	0.408

### Gastrointestinal Functions

As shown in Table 3, in comparison with the control group, fewer people with diarrhea were observed in the treatment group on the 7th day after the early bedside cycle exercise. In line with this finding, there were significantly more patients in the treatment group with increased bowel sounds than in the control group (*P* < 0.05). However, there were no significant between-group differences on the 1st, 4th, and 7th days after early exercise with respect to the number of patients with vomiting and gastric retention (*P* > 0.05) in these two groups.

**TABLE 3:** Gastrointestinal functions of patients in control and treatment group.

	The 1st day		The 4th day		The 7th day	
	N	%	N	%	N	%
**Diarrhea**						
Treatment group	15	28.8	10	19.2	6	12. 5
Control group	16	32	17	34	14	28
X^2^	0.12		2.857		4.382	
P	0.729		0.091		0.036*	
**Vomiting**						
Treatment group	3	5.8	5	9.6	2	3.8
Control group	3	6	5	10	4	8
X^2^	0		0		0.221	
P	1		1		0.638	
**Gastric retention**						
Treatment group	2	5.8	3	5.8	3	5.8
Control group	2	4	4	8	3	6
X^2^	0		0.003		0	
P	1		0.957		1	
**Increased bowel sounds**						
Treatment group	28	53.8	35	67.3	41	78.8
Control group	27	54	28	56	30	60
X^2^	0		1.38		4.28	
P	0.988		0.24		0.039*	

*p < 0.05 compared with control group.

### Nutrient Status

**FIGURE 3:**
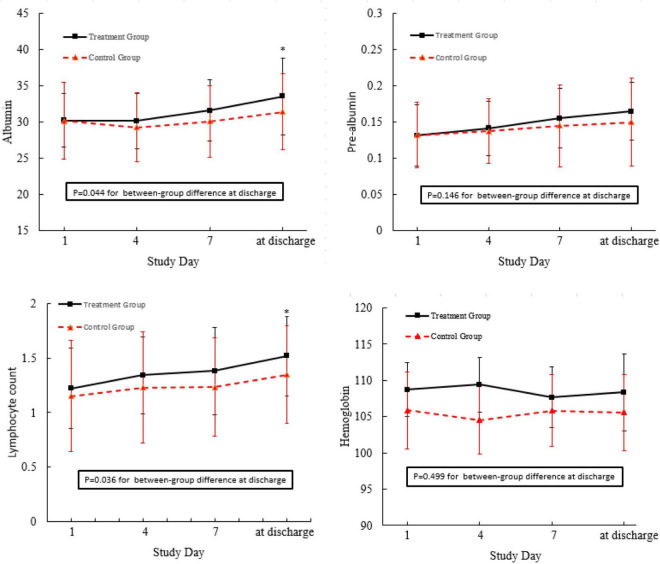
Nutrient status of the patients admitted to this trial. *p < 0.05 compared with control group.

In this field, there are limited studies on the lack of markers for measuring and assessing GI function as means of determining organ failure (32). Simple biochemical parameters such as albumin, hemoglobin, and TLC have long been identified as indicators of nutrient malabsorption associated with GI dysfunctions. As shown in Figure 3, in comparison with the control group, the albumin levels and lymphocyte count of the patients in the treatment group were significantly higher, while there were no significant between-group differences with respect to hemoglobin and prealbumin when the patients were discharged from the hospital.

### Other End Points

For those who had withdrawn from MV in both groups, the average time was 19.3 ± 7.7 days. The number of days free from MV differed significantly between the two groups [17 days (11.25–22 days) vs. 20 days (15.8–25.5 days), *P* = 0.036]. However, no statistically significant differences were observed in the length of ICU stay [27 days (20.3–33.8 days) vs. 29.5 days (22.3–40 days), *P* = 0.192] or hospital stay [36.5 days (29.3–44.8 days) vs. 37 days (26–51 days), *P* = 0.663] between the treatment and control groups, respectively (Table 4). All experimental subjects in these two groups were able to wean from MV at ICU discharge.

**TABLE 4:** Other end points of patients in the control and treatment group.

	Group	P50(P25∼P75)	*Z*	*p*
Days free from MV	Control	20(15.75–25.5)	2.095	0.036*
	Treatment	17(11.25–22)		
Length of ICU stay	Control	29.5(22.75–40)	1.306	0.192
	Treatment	27(20.25–33.75)		
Length of hospital stay	Control	37(26–51)	0.435	0.663
	Treatment	36.5(29.25–44.75)		

*p < 0.05 compared with control group.

## Discussion

Gastrointestinal failure is a well-known and common complication that influences adverse outcomes in critically ill patients (33). Pathophysiological abnormalities, including disturbances in motility and absorption, destruction of mucosal integrity, microbiological changes, increased intra-abdominal pressure, and potential alimentary tract infection, are the main underlying causes of GI dysfunctions and impairments in ICU patients (34, 35). It has been widely recognized that early and frequent exercise training prevents gastrointestinal dysfunction (36–38).

A bedside cycle ergometer is a stationary device designed to enable cycling revolutions of the arms and/or legs, and it can be prescribed to provide passive and active physical training (19, 21, 39). Recent literature reported that training with the cycle ergometer increased quadriceps strength, functional status recovery, and 6-min walking distance (6MWD) at the time of discharge (20). Historically, the large-scale use of this treatment was designed to improve the recovery of patients diagnosed with chronic obstructive pulmonary disease in outpatient settings (40, 41). Currently, a considerable number of researchers have also assessed its effects during hospitalization, particularly in ICU settings (31, 42). Published clinical trials related to exercise training in mechanically ventilated patients were within several weeks after the initial admission to the ICU (43). Nevertheless, many studies have explored whether GI disturbance might peak within the first week of an ICU stay (14, 44). This study suggests that exercise training as a therapeutic complement to prevent GI dysfunction should be initiated as early as possible.

Gastrointestinal symptoms and increased intra-abdominal pressure have been regarded as good signs for assessing gastrointestinal dysfunctions in patients with a critical illness (45, 46). Previous studies have suggested that the effects of motor exercise on enteric function are mixed (6, 40). Larson et al. demonstrated that there was no statistically significant difference between the groups in terms of gastrointestinal status after motor exercise (47). In the study conducted by Cheifetz et al. the researchers suggested that patients who underwent early mobilization were able to overcome many stressful obstacles that induced nausea or vomiting, pain, anorexia, tiredness, difficulty breathing, and the inability to defecate (48). The findings of this study suggest the positive impact of early bedside cycle exercise on GI system dysfunctions, such as vomiting, diarrhea, decreased bowel sounds, and gastric retention.

Several studies indicate an inverse relationship between physical activity and the risk of other gastrointestinal-related diseases, such as diverticular diseases, constipation, and cholelithiasis (49, 50). This is probably because regular, moderate exercise can accelerate gut transit of chyme and fecal residues and intraluminal gas (50). In this regard, light and moderate exercises are well tolerated and can benefit patients with bowel diseases (42). Although the benefits of an exercise regimen are widely documented, the underlying molecular mechanisms remain unknown. Evidence indicates that there are several potential mechanisms through which early exercise intervention has positive effects on GI abnormalities (51). The gut-associated lymphoid tissue is located throughout the small and large intestine and contains approximately 70% of the body’s immune cells. Several animal studies performed by Hoffman-Goetz et al. found that exercise alters the gene expression of intraepithelial lymphocytes, downregulates proinflammatory cytokines, and upregulates anti-inflammatory cytokines and antioxidant enzymes, which are essential for mediating GI functions (52–54). Similarly, exercise may impact the integrity of the gut mucus layer, which plays an important role in keeping gut microbes from adhering to the gut epithelium and thus improves the long-term resilience of the gut barrier. Exercise training also remarkably controls the mitochondrial dysfunction of the gut. Ali Khorjahani et al. found that gut mitochondrial dysfunction, which has been found to be closely linked with the pathophysiology of GI disorders, could be decreased *via* early exercise with a running wheel (55). Overall, mild-to-moderate intensity physical exercise has a protective effect on the gastrointestinal tract, but more research is needed to determine which of these mechanisms are responsible for the impact of exercise training on the adaptation of gut functions.

Intensive care unit patients who receive MV may experience nutrient malabsorption associated with GI dysfunctions and related treatments, which may result in malnutrition. Simple biochemical parameters, such as albumin, hemoglobin, and TLC have long been identified as risk indicators of malnutrition-related complications (29, 55). It is known that early physical activity prevents malnutrition. In the study conducted by Yoshihiro, serum albumin levels were significantly increased after long-term endurance training (56). Although Marcelo recently demonstrated that moderate physical exercise increases total leukocyte count, it was found that there was no difference in the hemoglobin, hematocrit, and red blood cell count between the trained and untrained groups (57). The results of this study found that nutrient parameters, such as prealbumin, albumin, and TLC, in these two groups did not show significant differences on the fourth and seventh days after early bedside cycle exercise. However, exercise training led to a significant increase in the levels of prealbumin and TLC in the training group on the day before discharge, which demonstrated that early bedside cycle exercise has a positive impact on the long-term effect of nutrient status instead of a short-term effect. Additionally, bedside cycle exercise has a positive impact on prealbumin levels but has no significant effect on albumin levels. The most likely reason for this is that the level of albumin is likely to be affected by nutritional status and also by inflammation and infection, which restricts its application as a detection marker for critically ill patients. Moreover, albumin has a long elimination half-life, which limits its usefulness in evaluating short-term changes in both energy and protein intake (57).

Therefore, the finding that early bedside cycle exercise has a positive impact on GI function and nutrition status is clinically meaningful. When analyzing these promising results, several limitations of this study should be taken into consideration. First, due to the time constraint, the findings of this study could not successfully identify a patient population with a single diagnosis or with the same disease stage. As a result, different kinds of diseases, even differences in different stages of the same disease, might be potential confounding factors related to GI dysfunctions that patients experience. Additionally, the initial study was small and was performed in one department of one hospital, which limited the generalizability of the research, which means that more extensive research is needed. A third limitation is that the treatment group received an additional 20 min of physical training in comparison with the control group. The findings of this study cannot entirely exclude the possibility that an additional 20 min of conventional physical mobilization training in the control group would have the same beneficial effects, especially if this standard treatment proved to have better possibilities of being upright. However, the reason that bedside bicycles were prescribed for patients with MV was that cycling offered a well-organized and targeted training stimulus.

## Conclusion

This randomized controlled trial implied that early bedside cycle exercise training can commence during ICU hospitalization in patients who receive MV. When instituted early in ICU survivors with prolonged ICU hospitalization, physical therapy may promote rehabilitation of gastrointestinal dysfunction and malnutrition and improve adverse clinical outcomes, including prolonged duration of MV at discharge from the hospital.

## Data Availability Statement

The raw data supporting the conclusions of this article will be made available by the authors, without undue reservation.

## Ethics Statement

The studies involving human participants were reviewed and approved by the Research Ethics Committee of First People’s Hospital of Kunshan and the Nanjing Medical University. The patients/participants provided their written informed consent to participate in this study. Written informed consent was obtained from the individual(s) for the publication of any potentially identifiable images or data included in this article.

## Author Contributions

TY provided the laboratory space, performed the experiments, prepared the figures, and wrote the manuscript. FC performed the experiments and interpreted the data. RJ designed the experiments, interpreted the data, wrote the manuscript, and directed the project. All authors read, discussed, and approved the final manuscript.

## Conflict of Interest

The authors declare that the research was conducted in the absence of any commercial or financial relationships that could be construed as a potential conflict of interest.

## Publisher’s Note

All claims expressed in this article are solely those of the authors and do not necessarily represent those of their affiliated organizations, or those of the publisher, the editors and the reviewers. Any product that may be evaluated in this article, or claim that may be made by its manufacturer, is not guaranteed or endorsed by the publisher.
